# Increased frequency of planetary wave resonance events over the past half-century

**DOI:** 10.1073/pnas.2504482122

**Published:** 2025-06-16

**Authors:** Xueke Li, Michael E. Mann, Michael F. Wehner, Shannon Christiansen

**Affiliations:** ^a^Department of Earth and Environmental Science, University of Pennsylvania, Philadelphia, PA 19104; ^b^Applied Mathematics and Computational Research Division, Lawrence Berkeley National Laboratory, Berkeley, CA 94720

**Keywords:** climate change, extreme events, planetary waves, Arctic amplification, ENSO

## Abstract

We demonstrate a tripling in the frequency of planetary wave resonance events over the past halfcentury, coinciding with the rise in persistent boreal summer weather extremes. This increase aligns with changes in the underlying climate conditions favoring these events, including amplified Arctic warming and land–sea thermal contrast. We also observe increased prevalence of resonant amplification events following the mature phase of strong El Niño events, suggesting that such events may precondition the mean state conditions in ways that favor large-scale quasi-stationary wave patterns and quasi-resonant wave amplification. Since the impact of anthropogenic warming on quasi-resonant amplification is not well captured by current-generation climate models, it is likely that models are underpredicting the potential increase, indicating even greater risk of persistent extreme summer weather events with ongoing warming.

## Significance

Climate change is increasing the frequency and severity of boreal summer extreme weather events, many of which are inextricably linked to the phenomenon of resonant planetary wave amplification. However, due to incomplete knowledge of climate change impacts on these dynamical mechanisms, combined with limited observational records, significant uncertainties remain regarding the magnitude of such an impact. Our analysis reveals a threefold increase in this phenomenon over the past seven decades, closely tied to amplified Arctic warming and land–sea thermal contrast. We also observe increased prevalence of resonance events during the mature phase of strong El Niño events. These findings advance our understanding of how both human-caused climate change and natural climate variability impact mid-latitude atmospheric dynamics and extreme weather events.

Climate change is increasing the frequency and severity of summer extreme weather events in the mid-latitudes of the Northern Hemisphere, from record-shattering heatwaves (1) to heavy precipitation and flooding (2), to prolonged droughts (3) and more extensive wildfires (4), as well as compounding effects due to interactions between extreme events (5). Continued warming is projected to magnify these trends (6, 7). While thermodynamic factors contribute to such extremes through shifts in mean temperature (8) or increases in atmospheric moisture content (9), assessing the role of dynamical factors remains challenging due to substantial natural variability and the uncertainty in the nature of the forced dynamical changes (10). The interplay between anthropogenic forcing and intrinsic climate variability introduces further complexities (11), rendering it fertile ground for continued exploration.

Over the past decade, a substantial body of literature has addressed how boreal summer extratropical extreme weather events are influenced by human-induced warming, particularly through the modulation of large-scale atmospheric circulation patterns (12–14). Some studies have focused on the weakening of storm tracks (15) and the jet stream (16). Other work (17, 18) has implicated the additional role of quasi-resonant amplification (QRA) of planetary waves, which favors highly persistent summer weather extremes through the resonance states of free synoptic-scale waves and forced planetary-scale waves. This phenomenon underlies major boreal summer weather extremes such as the 2003 European heatwaves (19), the simultaneous occurrence of the 2010 Russian heatwave and Pakistan flooding (20), the 2016 Alberta wildfire (21), and the 2021 Pacific Northwest heat dome event (22). While the precise role of anthropogenic climate change on observed trends in planetary wave resonance is still being debated in the literature (23, 24), analyses of the latest multimodel simulations suggest a likely role (25).

Two primary theoretical frameworks have been proposed for interpreting changes in boreal summer large-scale atmospheric circulation. On one hand, Arctic summer warming since 1979, though weaker than in other seasons, still exceeds the global average by a factor of two (26), resulting in the reduction of the meridional temperature gradient, decreased baroclinicity, and weakened upper-level zonal winds through the thermal wind relation (27). Some studies suggest that these changes tend to favor midlatitude stationary waves and enhance jet stream waviness (28, 29). However, the linkage between Arctic changes and the weakening of mid-latitude circulation remains a subject of ongoing debate (30, 31). On the other hand, while Arctic warming may decrease the meridional temperature gradient in the lower troposphere, shifting the jet stream equatorward, tropical warming could increase the gradient in the upper troposphere, potentially shifting it poleward (32, 33). This creates a “tug-of-war” effect on mid-latitude circulation responses. Additionally, anthropogenic aerosol emissions further influence these dynamics, with decreased aerosols over Europe leading to warming in mid-to-high latitudes, whereas increased aerosols over South and East Asia induce cooling in the tropics and subtropics (16). The phenomenon of QRA is potentially influenced by each of these competing effects.

The purpose of this research is to investigate the observed trend in QRA and interpret that trend in terms of the role of both Arctic and tropical warming. Our past work on the role of QRA has employed a fingerprint method that delineates QRA-favorable conditions based on lower atmospheric temperatures and the thermal wind relationship. This indirect approach has proven useful for assessing the phenomenon of QRA in current-generation climate models, whose wave dynamics remain inadequately resolved (34). The short duration of detailed upper-air measurements, however, proves challenging for assessing changes in QRA events directly (35, 36). Here, we make use of extended reanalysis data and compare both direct and indirect measures of QRA to demonstrate a robust and statistically significant trend in QRA activity tied to historical anthropogenic warming.

## Observed Changes in Wave Resonance Events

**Fig. 1.**
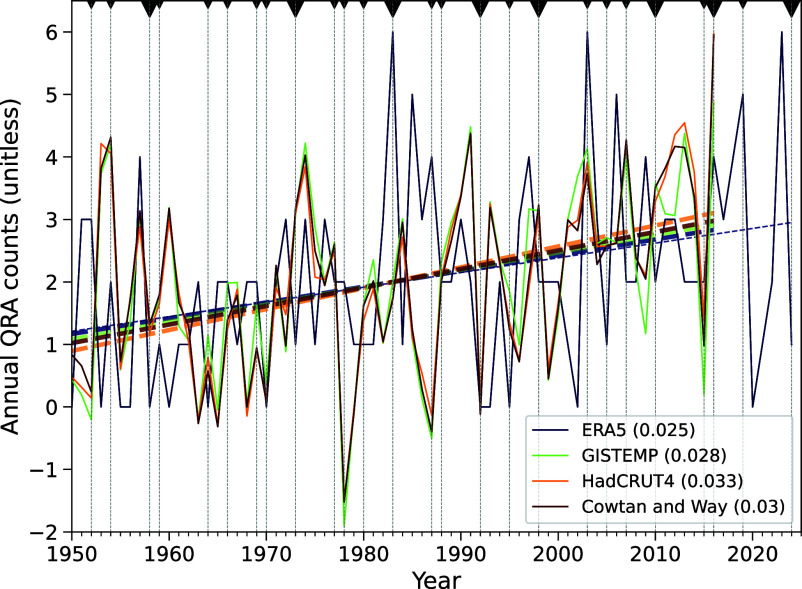
Time series of the boreal summer QRA event counts from 1950 to 2024. The annual QRA counts (unitless) based on ERA5 reanalysis data are represented by the blue line, while the series of QRA fingerprints (unitless)—an anomalous meridional temperature “fingerprint” associated with QRA-favorable conditions—using the three series of observational surface temperatures (GISTEMP, HadCRUT4, and Cowtan and Way datasets) are indicated by the green, orange, and red lines, respectively. The QRA fingerprint series (which are available from 1950 to 2016) are centered and rescaled to dimensionally match the annual QRA count series (Eq. **6** in *Materials and Methods*), allowing for direct comparison of the two series. The linear trends for each series during the period of overlap are depicted by thick dashed lines, with the respective slopes shown in brackets within the legend. The linear trend is also shown for the full QRA event count series through 2024 (thin dashed line). The trends are statistically significant at the *P* = 0.01 level using either a two-tailed Student’s *t* test or a nonparametric Mann-Kendall test (39) and the slopes are statistically indistinguishable. Gray vertical dashed lines denote the years when El Niño becomes mature based on the Oceanic Niño Index (ONI) (*Materials and Methods*). Strong to very strong events are represented by large triangles, while weak to moderate ones are indicated by small triangles (see also *SI Appendix*, Table S1).

We employed long-term ERA5 reanalysis data dating back to 1950 and applied the same QRA event detection criteria used in previous work (37, 38). A QRA event is counted as such if high-amplitude, quasi-stationary planetary waves of wave numbers 6, 7, or 8 are found in the atmospheric wind field in ERA5 data, and at the same time certain resonance conditions are met (see detailed description, including Eqs. **1**–**3**, in *Materials and Methods*). The annual QRA count series is calculated by summing the QRA episodes within each year. Using the full seven decades of data, a statistically significant positive trend emerges in the annual count of boreal summer QRA events, with particularly notable occurrences in the recent decade, corresponding roughly to a tripling from ~1 event/y to ~3 events per year over the past 70 y (Fig. 1).

The tendency for larger positive values during the post-1980 period relative to the pre-1980 period raises the possible concern of data inhomogeneity, as that timing aligns with the introduction of new satellite instruments in the observing system in 1979 (40). However, a breakpoint analysis (41) reveals no statistical evidence for a discontinuity at 1979 (*SI Appendix*, Fig. S1). This finding is consistent with previous analyses suggesting an absence of discontinuities in the ERA5 product (42, 43).

More significantly, there are independent, indirect measures of QRA changes that show remarkably similar trends. We employed independent QRA fingerprint series derived from in situ instrumental surface temperature products (i.e., GISTEMP, HadCRUT4, and Cowtan and Way datasets) as presented by Mann et al. (17)(18) (see detailed description, including Eqs. **4** and **5**, in *Materials and Methods*). As a simple proxy for QRA events based solely on a zonal-mean temperature pattern (the fingerprint), the QRA fingerprint has been found to be associated with QRA-favorable conditions (17, 18, 25)—characterized by the presence of a steep and narrow subtropical jet and a double-jet structure in the zonal mean zonal wind. The QRA fingerprint is a probabilistic predictor of QRA-favorable conditions rather than a direct measure of annual event counts. As such, we might not expect it to capture the precise annual counts (which represent seasonal averages of a small number of events) but, rather, the interannual and longer-term variations in QRA behavior. The consistency of the positive trends across these three independent fingerprint series (green, orange, and red dashed lines in Fig. 1; each is statistically significant at the *P* = 0.01 level) with that of the reanalysis-derived QRA series provides an internally consistent picture of increasing QRA counts that is tied to the meridional pattern of surface warming.

**Fig. 2.**
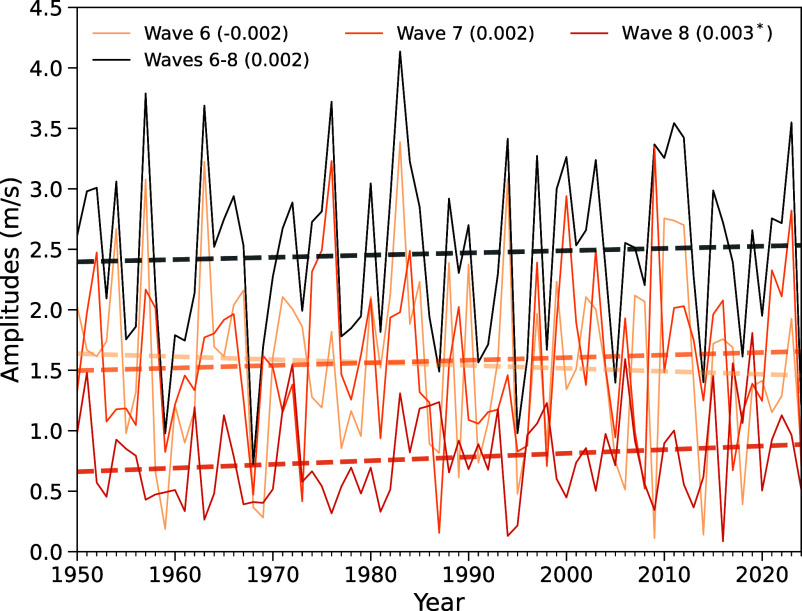
Time series of amplitudes for zonal wavenumbers k = 6, 7, 8 individually and for the entire band 6 to 8 from 1950 to 2024. Linear trends for each time series are shown as thick dashed lines, with the corresponding slopes indicated in brackets within the legend. A single asterisk (*) indicates statistical significance at the 95% confidence level.

As QRA events are characterized by simultaneous amplitude and phase characteristics (i.e., the requirement of quasi-stationary behavior which is tied to near-zero phase velocity), it is of interest to investigate whether the observed rise is associated with increases in either the amplitude or incidence of waves in the k = 6 to 8 band or, rather, increased prevalence of the joint phase and amplitude conditions supporting QRA behavior. We performed such an analysis over the full available period 1950–2024 (Fig. 2). Consistent with previous work analyzing the much shorter time period of 1979–2012 [Petoukhov et al. (44), in response to Screen and Simmonds (45)], we observe only a very weak (and statistically insignificant) trend in wave amplitudes in the k = 6 to 8 band overall. Interestingly, we do identify a statistically significant (*P* = 0.05) increase in the amplitude of the highest wavenumber (k = 8) planetary waves and a small increase for k = 7, which is offset by a small decrease for k = 6. These observations hint at the possibility of changing amplitudes within the broader k = 6 to 8 band that favor higher wavenumber/shorter wavelength resonant planetary waves, but they do not show evidence for increased wave amplitudes of resonant waves overall. An additional analysis reveals a similarly weak (and statistically insignificant) trend in the annual incidence of planetary waves overall in the k = 6 to 8 band (*SI Appendix*, Fig. S2). We conclude that the substantial observed increase in QRA counts is driven not by increases in wave activity but, rather, with increases in the joint occurrence of wave amplitude and phase conditions that support QRA.

## Connection with ENSO

The El Niño/Southern Oscillation (ENSO) can induce anomalies in extratropical circulation that contribute to extreme events (46). These anomalies manifest through equatorward (poleward) shifts of the upper-level jet stream during El Niño (La Niña) events (47, 48), and the generation of large-scale atmospheric Rossby waves that propagate into the extratropics (49, 50). The magnitude of the extratropical response can be amplified when the climatological-mean stationary wave pattern is forced by the land–sea thermal contrast and major mountain ranges (51). Despite the known influence of ENSO on jet stream behavior and Rossby wave propagation, the connection between the evolution of the ENSO cycle and QRA characteristics remains largely unexplored, perhaps because ENSO teleconnections tend to be weaker during the boreal summer (52).

Interestingly, peak QRA years evident in the reanalysis data often follow the mature phase of strong El Niño events (*SI Appendix*, Table S1). To explore this relationship further, we analyzed the correlation between the boreal winter Oceanic Niño Index (ONI) of El Niño and subsequent boreal summer QRA counts. The ONI, a measure of ENSO, represents the 3-mo running mean of sea surface temperature (SST) anomalies in the Niño 3.4 region. El Niño events are defined as periods when the ONI exceeds a threshold of +0.5 °C for at least five consecutive 3-mo running mean of SST anomalies (*Materials and Methods*). The boreal winter ONI of a given El Niño event corresponds to the 3-mo running mean spanning December of the development year and January to February of the decay year, capturing the mature phase of the El Niño. Our findings reveal a significant positive correlation (*r* = 0.63, one-sided *P* = 0.07) for strong to very strong El Niño events (ONI ≥ 1.5 °C), providing quantitative evidence that an increased prevalence of QRA events tends to follow strong El Niño conditions, and often during the transition from strong El Niño to La Niña. This phase relationship aligns with previous work showing significant increases in heat extremes during the decaying summer of El Niño events (53–55). The potential for a stationary circumglobal teleconnection (CGT) wave pattern excited by ENSO tropical forcing during the boreal summer has been established in previous work (56, 57). Diabatic heating associated with tropical heating anomalies serve as a source of upper-tropospheric perturbations that project onto the midlatitude waveguide (53), leading to the development of the CGT pattern and contributing to the formation of a double-jet structure. This configuration provides the necessary latitudinal waveguide via a confined subtropical jet (58), effectively trapping Rossby waves and favoring resonance events (50). Notably, Rossby wave propagation into the mid-latitudes tends to strengthen during the transition from El Niño to La Niña (59), further amplifying the potential for such quasi-resonant behavior.

Not all years with peak QRA counts, of course, correspond to strong El Niño events. Various sources of interannual variability, including external factors (e.g., volcanic forcing) and internal dynamics [e.g., the North Atlantic Oscillation (NAO—see ref. 60)], can influence QRA occurrences. Nevertheless, a power spectral density analysis of the QRA time series reveals prominent and statistically significant spectral peaks within the interannual (2 to 7 y) band associated with ENSO (*SI Appendix*, Fig. S3). It is additionally noteworthy that the QRA fingerprint series do not capture the El Niño-related peaks seen in the reanalysis-based proxy QRA series, suggesting certain QRA influences that are not well captured by the approximations implicit in the fingerprint approach.

## Connection with Temperature Patterns

**Fig. 3.**
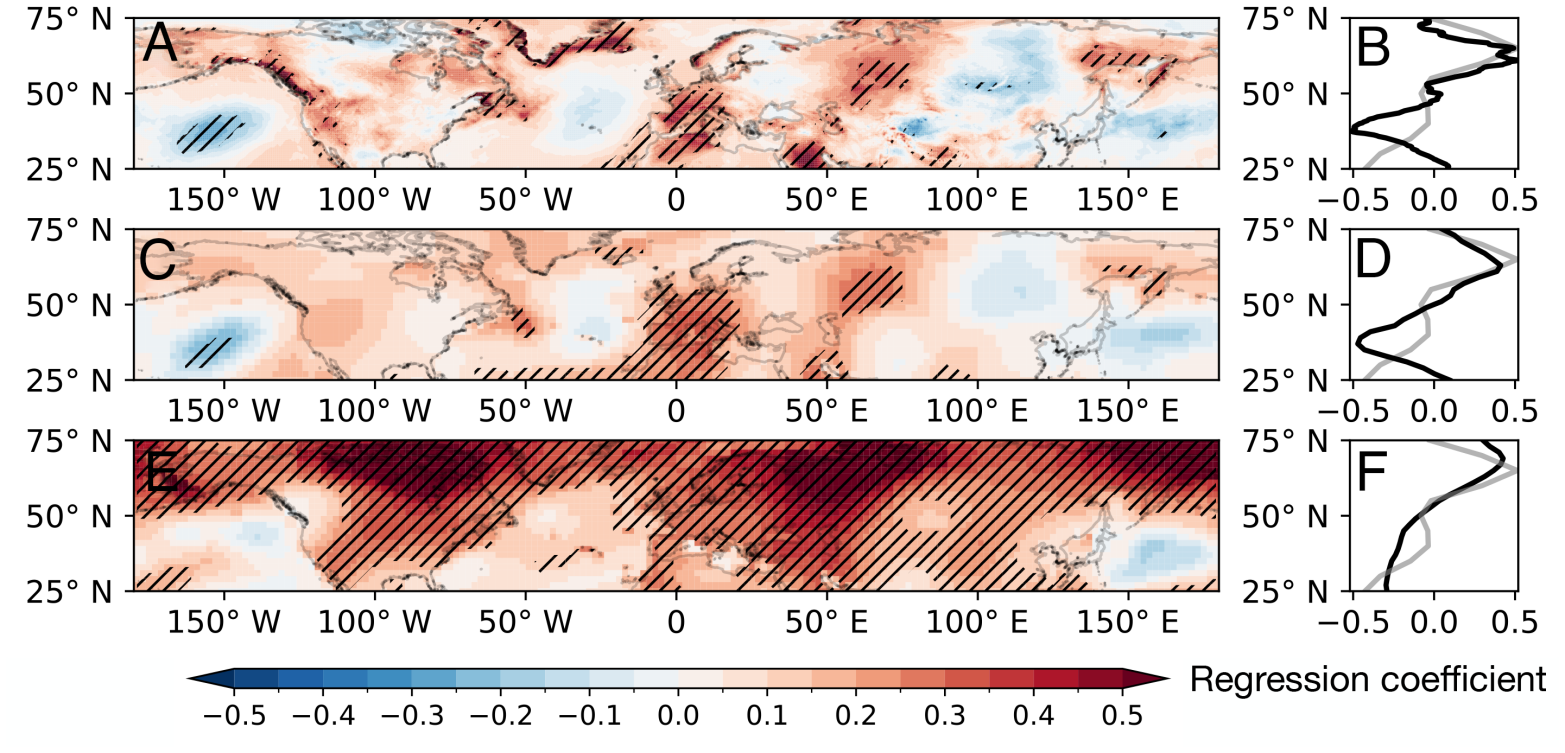
Spatial patterns of the boreal summer surface temperature anomalies in relation to indices indicative of QRA events or QRA-favorable conditions. The regression coefficients (*K* per event) are computed for (*A*) ERA5 surface temperature anomalies with the reanalysis-based QRA count series, (*C*) GISS surface temperature anomalies with the reanalysis-based QRA count series, and (*E*) GISS surface temperature anomalies with the GISTEMP QRA fingerprint series for the period 1950−2016. Hatching indicates significance at the 95% confidence level. Their zonal averages as a function of latitude are represented by the black line in (*B*, *D*, and *F*), respectively, while the gray line indicates the QRA zonal fingerprint centered on zero, similar to figure 1C of ref. 18. For visual comparison, the means of the black lines are shifted so that they have the same mean as the gray lines. The region of interest is confined to the mid-latitudes (25°N–75°N).

To better elucidate the large-scale surface temperature patterns typically associated with annual QRA counts, we conducted regressions of surface temperature anomalies (*SI Appendix*, Fig. S4 for the trend of surface temperature anomalies) onto both the reanalysis-based QRA count series (Fig. 3 *A*–*D*) and the QRA fingerprint series (Fig. 3 *E* and *F*) from Fig. 1. In both cases, a consistent pattern of positive anomalies is observed over high latitudes, which closely mirrors the QRA zonal fingerprint (Fig. 3 *B*, *D*, and *F*), emphasizing the role of amplified Arctic warming, particularly over land areas experiencing a rapid decline in spring snow cover (28, 61). There are some differences in the spatial details, however, with greater magnitudes of warming evident in the QRA fingerprint series over the Northern Pacific, northern Canada, and Western Eurasia, the spatial pattern of which closely resembles the subset of climate projections exhibiting the most positive QRA-trending behavior, as shown in ref. 17. This stronger relationship is invariant with respect to data source (e.g., Fig. 3 *A* vs. *E*). In both cases, we see a distinct land-ocean contrast, but it is particularly conspicuous in the reanalysis-based QRA count series, with noteworthy land-ocean gradients off the Pacific coasts of North America and Eurasia. A *t* test indicates that the differences in their zonal profiles are not statistically significant in the mid-latitudes. A tendency toward a wave 7-like pattern in the reanalysis-based QRA count series (Fig. 3 *A* and *C*) aligns with findings from ref. 23, suggesting a higher tendency for phase locking behavior of wave 7, associated with heat events over Western Europe and a “cold blob” over the Atlantic (24). The comparatively subdued response pattern for the QRA fingerprint series suggests that the thermal wind assumption used to relate lower atmospheric temperatures to upper-level zonal winds may not fully capture the totality of mechanisms influencing QRA. The discrepancy is particularly evident in regions where zonal temperature gradients, created by amplified land warming, may play a role in phase-locking (23), likely acting as stationary vorticity sources that initiate and sustain atmospheric waves (50).

## Implications of Climate Change for the Quasi-Resonance Phenomenon

Our analysis informs our understanding of how human-caused climate change is impacting summer extreme weather events. An increase in resonant planetary wave events, which favor persistent summer weather extremes, is evident since the mid-twentieth century, with an increase from roughly 1 to 3 events per summer on average. This increase is evident from both direct and indirect measures of QRA activity. The implicated mechanisms include amplified summer Arctic surface warming and increases in land–sea thermal contrast (*SI Appendix*, Fig. S5) favoring resonant planetary wave behavior, both of which have been linked to anthropogenic climate change.

In the context of amplified Arctic warming, the mean jet stream becomes narrower as the pole-to-equator temperature gradient weakens (13, 62), occasionally facilitating the formation of circumglobal, zonally oriented wave patterns by acting as the waveguide, trapping free synoptic-scale waves that resonate with orographic and thermally forced planetary-scale waves (37). The phenomenon generates enduring high-pressure ridges and low-pressure troughs, favoring persistent weather extremes. Past work analyzing historical simulations reveals a consistent increase in QRA-favorable conditions with amplified Arctic warming (17, 18). The rapid loss in spring snow cover, which enhances drying and warming of high-latitude landmasses and the reduction in summer Arctic sea ice, may play a role in summer Arctic amplification. Future projections, on the other hand, show substantial dependence on projected changes in aerosol forcing linked to indirect aerosol effects; models with especially large indirect aerosol effects often exhibit Arctic deamplification and muted increases in inferred QRA activity (17).

One important caveat in any future projected behavior involves ENSO. Asymmetrical heating anomalies associated with tropical convective activity, such as those arising from the ENSO, can also drive the amplification of waveguide teleconnections during boreal summer (56). During the historical era, as noted in this study, an increased prevalence of QRA events has been observed following strong El Niño events. Different phases of ENSO are likely to trigger quasi-stationary Rossby waves from distinct source regions, with varying propagation arc and wave structures and impact centers (24). For example, one study (63) has found that the circumglobal wave train, characterized by a zonal wavenumber-5 structure, is closely associated with suppressed tropical Pacific convection. Given the substantial uncertainty in current-generation climate models regarding changes in the amplitude and frequency of El Niño events with projected anthropogenic climate change (64), there is accordingly additional uncertainty in future projections of QRA and associated persistent summer weather extremes. Further research is needed to achieve a more thorough mechanistic understanding of the factors impacting interannual and longer-term variability in QRA, but it is clear that there is a trend toward more frequent QRA events during the modern historical era that is likely tied to anthropogenic climate change.

## Materials and Methods

### QRA Detection Scheme.

Theoretically, QRA builds on the classical work that demonstrated the potential for resonance in stationary planetary waves (65) and waveguide-like behavior in these waves (50, 66). Expanding on this, Petoukhov, Rahmstorf, Petri and Schellnhuber (37) and Petoukhov et al. (67) demonstrated that planetary waves within synoptic wave numbers 6 to 8 can become effectively trapped in a latitudinal waveguide, depending on the meridional profile of the midlatitude westerly jet, thus providing a mechanism for the time dependence of resonant behavior. If these waveguide conditions hold—confining waves meridionally and preventing the dispersion of wave energy—then a pronounced amplification of waves excited by orographic or thermal forcing can occur, leading to hemispheric-wide, persistent high-amplitude wave patterns and their associated anomalous surface weather in the mid-latitudes.

Mathematically, the QRA detection scheme involves identifying the formation of a waveguide and the effective forcing pattern favorable for quasi-resonance to occur. A more detailed description of the quasi-resonance mechanism and its detection can be found in refs. 37 and 38, with a brief overview provided here. Starting with the formation of a zonally oriented waveguide that can confine synoptic-scale free waves with zonal wave numbers k ≈ 6 to 8, this waveguide is bounded by two turning points (TPs) where the square of the meridional wave number $l^{2}=0$ at each TP and $l^{2}> >0$ in between while the zonal mean zonal wind $\bar{u}> >0$ in between and in the vicinity of the TPs. This configuration traps quasi-stationary free waves, preventing their dissipation toward the poles or the equator. Occasionally, a double westerly jet behavior is observed, featuring a strong and narrow jet positioned on the subtropical flank and another on the poleward flank. $l^{2}$ is a function of $\bar{u}$, latitude φ, and the zonal wave number k, which is derived by solving the linearized quasi-geostrophic barotropic vorticity equation under the Wentzel–Kramers–Brillouin (WKB) approximation at the equivalent barotropic level (EBL) for quasi-stationary free plane wave solutions, as governed by

[1] $$l^{2}=f\left(\bar{u},\varphi ,k\right)=\frac{2\Omega \;\;{\text{cos}}^{3}\;\varphi }{a\bar{u}}-\frac{{\text{cos}}^{2}\varphi }{a^{2}\bar{u}}\frac{d^{2}\bar{u}}{d\varphi^{2}}+\frac{\text{sin}\varphi \;\;\text{cos}\varphi }{a^{2}\bar{u}}\frac{d\bar{u}}{d\varphi }+\frac{1}{a^{2}}-{\left(\frac{k}{a}\right)}^{2},$$

where Ω is Earth’s rotational angular velocity and a is Earth’s radius.

If the waveguide is (almost) circumglobal, wave energy becomes effectively trapped, allowing waves to constructively interfere with the forcing, resulting in resonance and the amplification of forced waves within the zonal wave number range m = 6, 7, and 8. The corresponding amplitude is determined by

[2] $${\tilde{A}}_{m}=\frac{{\tilde{A}}_{\mathrm{eff}}}{\sqrt{{\left[{\left(k/a\right)}^{2}-{\left(m/a\right)}^{2}\right]}^{2}+(L/a^{2}+R^{2}/L{)}^{2}{\left(m/a\right)}^{2}}},$$

where L and R represent the characteristic Rossby radius and Rossby number for the eddies contributing effectively to the atmospheric near-surface and internal “eddy function”; and ${\tilde{A}}_{\mathrm{eff}}$ is the effective forcing amplitude (in m^−1^ s^−1^) derived by applying a zonal fast Fourier transformation (FFT) to the area-weighted meridional mean of the combined orographic and thermal forcing, $F_{\mathrm{eff}}$, at 300 hPa over the latitudinal range of 37.5°N–57.5°N. The forcing term $F_{\mathrm{eff}}$, given by the equation below, is calculated using the azonal temperature at 300 hPa ($T^{\prime }$) and the orography ($h_{\mathrm{orog}}$) regridded to a coarse resolution of 10° × 15° as described in ref. 68.

[3] $$F_{\mathrm{eff}}=\frac{2\Omega \;\;\sin\varphi \;\;\cos^{2}\varphi }{aT_{c}}\frac{\partial T^{\prime }}{\partial \lambda }-\frac{2\Omega \;\;\sin\;\varphi \;\;\cos^{2}\varphi }{\mathrm{aH}}\kappa \frac{\partial h_{\mathrm{orog}}}{\partial \lambda }$$

where $T_{c}$ is a constant reference temperature at the EBL, λ is longitude, H is the characteristic scale of the troposphere height, and κ is the characteristic value of the ratio of the $\bar{u}$ at 300 hPa to that at the mean orographic height.

The observed wave amplitude is computed by applying a zonal FFT to the meridional wind at 300 hPa averaged over 37.5°N–57.5°N. It is then used in the amplitude test, which is considered satisfied when it falls within the computed range of ${\tilde{A}}_{m}$ for values of k close to m, ensuring that ${\tilde{A}}_{m}$ is sufficiently large to indicate strong amplification. Here, k=m±0.2 is applied following the approach in ref. 38 and the rationale provided in ref. 67.

Additional criteria for constraining the waveguide and effective forcing amplitude to meet specific requirements are detailed in table 1 of ref. 38. This approach enables the identification of each day where QRA is or is not present. The annual count of QRA events is then calculated by summing the QRA episodes within each year. We refer to the whole series of annual QRA event counts from 1950 to 2024 as the QRA count series. It is important to note that the linearized assumption and the treatment of thermal forcing terms (35, 45), as outlined in theoretical formalism, may lead to situations where a waveguide is detected but waves are not effectively trapped. Therefore, it is essential to interpret the results with caution–QRA detection can only serve as a diagnostic tool rather than a predictive one (38).

We implemented the aforementioned methodology on daily mean fields of temperature, meridional winds, and zonal winds at 300 hPa during boreal summer (June–July–August, JJA) over the period 1950 to 2024. For this analysis, we utilized data from the European Centre for Medium-Range Weather Forecasts (ECMWF) Reanalysis v5 (ERA5), provided on a 2.5° horizontal grid. To filter out fast-traveling free Rossby waves, a 15-d running mean was applied. It is worth noting that while the absence of satellite observations prior to the 1970s may introduce some uncertainty into ERA5 estimates (69), the extension of the dataset back to the 1950s, when the number of upper-air observations increases around 1946 (70), is generally considered trustworthy for upper-air temperature and wind fields (at levels below 10 hPa) (43), which forms the basis of our analysis. The observed trend in QRA event counts is robust with respect to the precise criteria for detection, holding for both the least and most stringent conditions (*SI Appendix*, Fig. S6).

### QRA Fingerprint.

Given the higher uncertainty associated with climate models in representing planetary wave dynamics [e.g., meridional gradients in zonal wind $\bar{u}$ as per Eq. 1 where errors can reach as high as 344% in CMIP5 models (17) and 285% in CMIP6 (25) after two successive differentiations] compared to their ability to capture global patterns of surface temperature (34), Mann et al. (17, 18) developed a temperature-based fingerprint for QRA conditions to be reliably applied to state-of-the-art climate models. The QRA fingerprint relies on the meridional surface temperature profile T(φ), employing the thermal wind mechanism to infer the relationship between meridional surface temperature gradients and the change in upper-level zonal winds (Δu), expressed as

[4] $$\Delta u=-\frac{R}{f}\text{ln}\left(\frac{p_{s}}{p_{300}}\right)\frac{\mathrm{dT}}{d\varphi },$$

where R is the gas constant for dry air, f is the Coriolis parameter, $p_{s}$ is the surface pressure (set here as $p_{s}$ = 1,000 hPa), and $p_{300}$ is the pressure level at 300 hPa.

The QRA fingerprint, denoted as ${\mathrm{QRA}}_{\mathrm{fingerprint}}$, is thus defined as the anomalous temperature profile associated with QRA, that is, the zonal mean temperature profile averaged over QRA events, $T_{\mathrm{QRA}}\left(\varphi \right)$, minus the climatological zonal mean temperature profile, $T_{\mathrm{clim}}\left(\varphi \right)$ (see the gray plot in Fig. 2 and Eq. **4**). With this definition, it is important to interpret the QRA fingerprint with caution: While fingerprints are valuable for analyzing long-term trends, they may be less effective at capturing individual seasonal totals, which are the sum of a small number of events.

[5] $${\mathrm{QRA}}_{\mathrm{fingerprint}}=T_{\mathrm{QRA}}\left(\varphi \right)-T_{\mathrm{clim}}\left(\varphi \right).$$

For our analysis, we used boreal summer (JJA) observational surface temperatures from the Goddard Institute for Space Studies Surface Temperature Analysis version 4 (GISTEMP v4; 1880–2016) (71), the Hadley Centre/Climatic Research Unit gridded surface temperature dataset 4 (HadCRUT4; 1850–2016) (72), and Cowtan and Way (1850–2016) (73), spanning the overlapping period 1950–2016, which is sufficiently long to capture the relationships of interest, even if it does not extend—in this particular case—up to the present. Consistent with prior research (25), our analysis focused on long-duration wave number 6 to 8 events lasting for 10 d or more to define periods conducive to QRA conditions. Additionally, we limited the zonal mean profiles to the mid-latitude range of 25°N–75°N with a 5° interval.

To align the QRA fingerprint series with the reanalysis-based QRA count series to facilitate a more direct and comparable interpretation, ${\mathrm{QRA}}_{\mathrm{fingerprint}}$ is mean-centered and rescaled (${\mathrm{QRA}}_{fingerprint\_rescaled}$) to match the mean and SD of the reanalysis-based QRA count series. The equation for this rescaling is provided below.

[6] $${\mathrm{QRA}}_{fingerprint\_rescaled}=\left(\frac{{\mathrm{QRA}}_{\mathrm{fingerprint}}-\mu_{\mathrm{fingerprint}}}{\sigma_{\mathrm{fingerprint}}}\right)*\sigma_{\mathrm{actual}}+\mu_{\mathrm{actual}},$$

where $\mu_{\mathrm{fingerprint}}$ and $\sigma_{\mathrm{fingerprint}}$ are the mean and SD of the QRA fingerprint, and $\mu_{\mathrm{actual}}$ and $\sigma_{\mathrm{actual}}$ represent the mean and SD of the reanalysis-based QRA count series, respectively.

### Comparison of Observational Surface Temperatures.

An overview of the most widely used surface temperature datasets, including GISTEMP v4, HadCRUT4, and the Cowtan and Way dataset, is provided in ref. 74. While there is broad agreement among these datasets regarding the annual global surface temperature anomaly, a key distinction lies in their treatment of incomplete station coverage, particularly in the Arctic. The HadCRUT4 dataset avoids any form of interpolation, preserving direct traceability to observational records, though it sacrifices spatial coverage, particularly in areas like the Arctic where stations are sparse (72). In contrast, Cowtan and Way (73) employ kriging interpolation to reconstruct gridded temperature values with near-global coverage, emphasizing the importance of interpolation in capturing Arctic amplification. GISTEMP applies the most extensive interpolation, using a 1,200-km radius of influence for each station, which can potentially lead to overestimation (71) or underestimation (75) of Arctic warming, despite its good performance in capturing trends and correlations of global mean surface temperature, as independently validated by the Atmospheric Infra-Red Sounder (AIRS) (75). These differences in methodological approaches, combined with other factors such as station uncertainty and systematic biases, underscore the need for making use of multiple surface temperature datasets to ensure that the sensitivity of results to the specific methods used in dataset construction can be properly evaluated.

### Defining El Niño Events.

In this study, ENSO is quantified using the Oceanic Niño Index (ONI) calculated within the Niño 3.4 region (5°N–5°S, 120°–170°W), based on a running mean of sea surface temperature (SST) anomalies over a 3-mo period (76). An El Niño event is defined when the ONI surpasses a threshold of +0.5 °C for a minimum of five consecutive 3-mo running mean of SST anomalies. We specifically focused on the mature phase of El Niño during boreal winter. A total of 26 El Niño episodes are identified and listed in *SI Appendix*, Table S1, aligning closely with those diagnosed using alternative criteria such as historical Niño 3 and Niño 4 SST indices (77), or based on a bimonthly Multivariate El Niño/Southern Oscillation (ENSO) index (MEI.v2) (78). 13 out of 26 El Niño onset years coincide with years of high QRA counts. A Monte Carlo analysis demonstrates that this association is statistically significant at the one-sided *P* = 0.02 level. El Niño events are further categorized into weak, moderate, strong, and very strong based on the ONI values falling within the ranges of 0.5 to 0.9, 1.0 to 1.4, 1.5 to 1.9, and ≥2.0, respectively. Strong to very strong El Niño events are highlighted in bold in *SI Appendix*, Table S1, many of which have been extensively documented in prior research (79), including occurrences in 1972–73, 1982–83, 1997–98, and 2015–16.

### Monte Carlo Analysis.

To assess the statistical significance of the observed overlap between high-frequency QRA event years and El Niño development years in *SI Appendix*, Table S1, we conducted a Monte Carlo analysis. Specifically, we randomly selected the same number of high-frequency QRA event years and assigned them to random years within the observed period (1950–2024) and then counted how often these randomly assigned years coincided with El Niño development years. This process was repeated 10,000 times to generate a distribution of expected overlaps under a random scenario, providing a benchmark for comparison with the observed overlaps.

### Defining Boreal Summer Arctic Amplification Index and Land–Sea Thermal Contrast.

To better understand the underlying causes for the observed increase in QRA events, we examine long-term changes in the magnitude of Arctic warming and land–sea thermal contrast during boreal summer (JJA). Following previous work (17, 18, 25), Arctic Amplification index is defined as the JJA zonal mean surface temperature difference between 65–75°N and 25–60°N. Land–sea thermal contrast is calculated as the difference between land and ocean temperature anomalies over the Northern Hemisphere (80), averaged for the boreal summer period. In both cases, temperature anomalies are computed with respect to the 1950–2024 climatological means.

### Statistical Analyses and Significance Test.

Linear least-squares regression was employed to assess trends of time series. Statistical significance was assessed based on both Student’s *t* test and a nonparametric Mann-Kendall trend significance test.

We also used least-squares regression to compute regression coefficients between QRA count (or fingerprint), referred to as QRA, and surface temperatures, denoted as $T_{s}$, as described below

[7] $$T_{s}=\alpha *QRA+\varepsilon ,$$

where $T_{s}$ is obtained from GISTEMP v4 with a 1,200 km smoothing or from ERA5 with a 0.25° spatial resolution; α and ε are the regression slope and residual, respectively. To improve representativeness and ensure comparability, QRA is normalized at each grid box to have unit variance. The slope coefficients are mapped in Fig. 2, representing the fractional change in the $T_{s}$ per unit fractional change in QRA.

### Breakpoint Analysis.

Data inhomogeneities can introduce mean-state biases, discontinuities, or spurious trends (81). The ERA5 reanalysis addresses such uncertainties by assimilating multiple data sources and applying a physics-based model to minimize unrealistic values. Despite these improvements in accuracy, possible temporal inhomogeneity may still exist due to biases in the model or observations or from intermittent changes in observational input, affecting long-term temporal consistency and the reliability of trend analysis. This issue is particularly pronounced when a substantial volume of satellite observations began to be assimilated into the system around 1979 (42).

To examine the presence of any potential discontinuities in the ERA5 reanalysis results that could influence our main findings regarding the increased trend in QRA events from 1950 onward, we conducted a breakpoint analysis (41). This analysis calculates breakpoints in the linear regression relationships by minimizing the residual sum of squares (RSS) and determining the optimal number of breaks that result in the minimum Bayesian information criterion (BIC) partition (82). The significance of this analysis is evaluated using structural change tests based on *F* statistics within linear regression models. We reject the null hypothesis of no structural change at the 95% significance level for a *P*-value is less than 0.05.

## Supplementary Material

Appendix 01 (PDF)

## Data Availability

The ERA5 reanalysis data are available at https://cds.climate.copernicus.eu/datasets/reanalysis-era5-pressure-levels?tab=overview (83). All codes and data used to generate the main figures are publicly accessible on GitHub at https://github.com/mann-research/XL-PNAS/ (84). All other data are included in the article and/or *SI Appendix*.
