# Supplementary material for: Increased frequency of planetary wave resonance events over the past half-century
Source: Proc Natl Acad Sci U S A. 2025 Jun 16;122(25):e2504482122. doi: 10.1073/pnas.2504482122 (PMC12207419; doi:10.1073/pnas.2504482122)
Supplement: Supplementary file 1 — Appendix 01 (PDF) [file pnas.2504482122.sapp.pdf]

Supporting Information for
Increased frequency of planetary wave resonance events over the past half-century

Xueke Li[1], Michael E. Mann[1], Michael F. Wehner[2], Shannon Christiansen[1]

* Corresponding author: Xueke Li and Michael E. Mann.
Email: xuekeli@sas.upenn.edu or mmann00@sas.upenn.edu

This PDF file includes:

Figures S1 to S6
Table S1

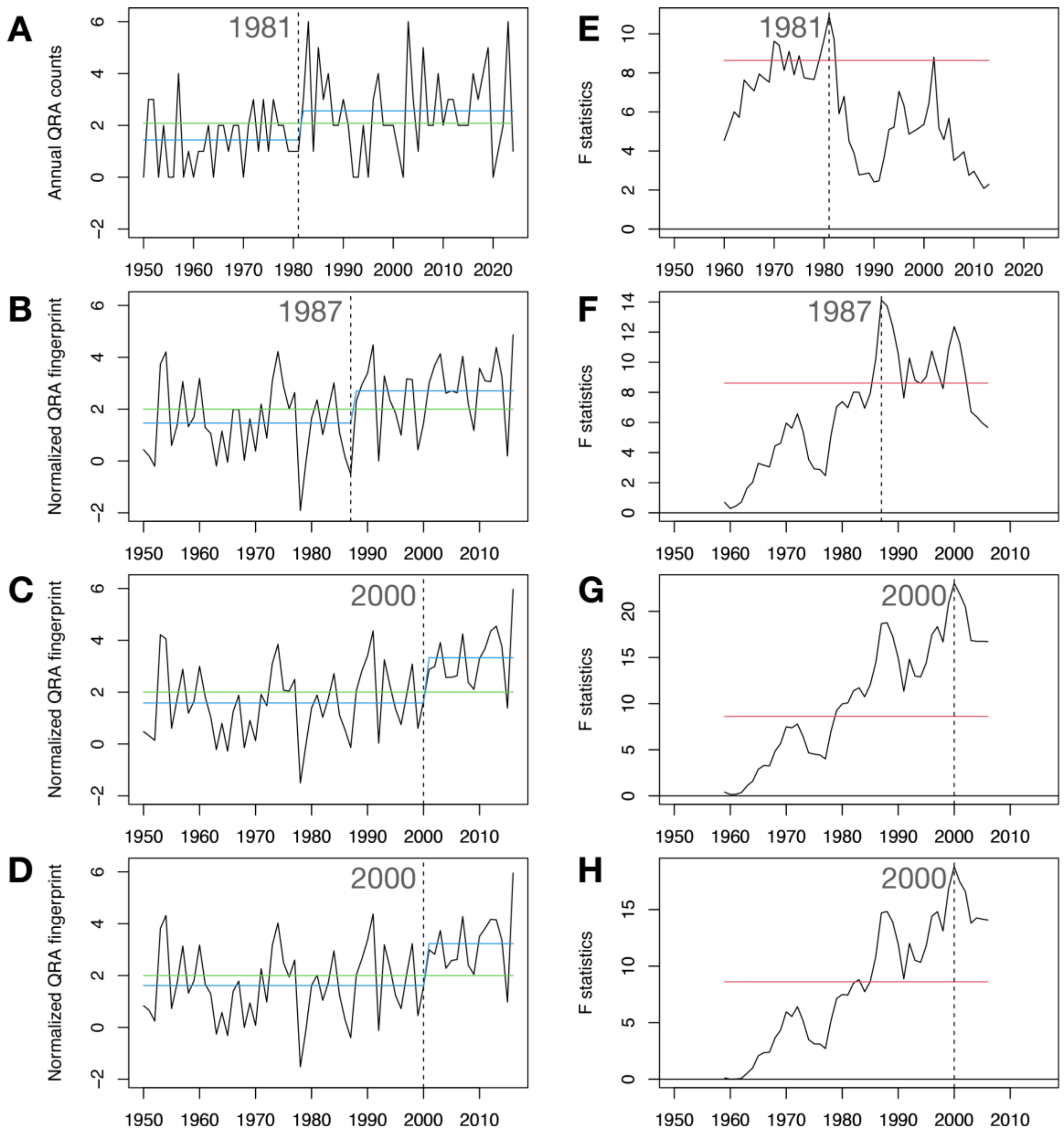

Fig. S1. Breakpoint analyses of four QRA series. (A) Reanalysis-based QRA event series spanning from 1950 to 2024. (B-D) QRA fingerprint series (1950–2016) based on GISTEMP, HadCRUT4, and Cowtan and Way surface temperature datasets, respectively. (E-H) Corresponding structural change tests based on *F* statistics. The vertical dashed line indicates the breakpoint. The green horizontal line represents the mean of the entire time series, while the blue line represents the mean of each segment partitioned by the breakpoint. The horizontal red line indicates the 95% confidence level.

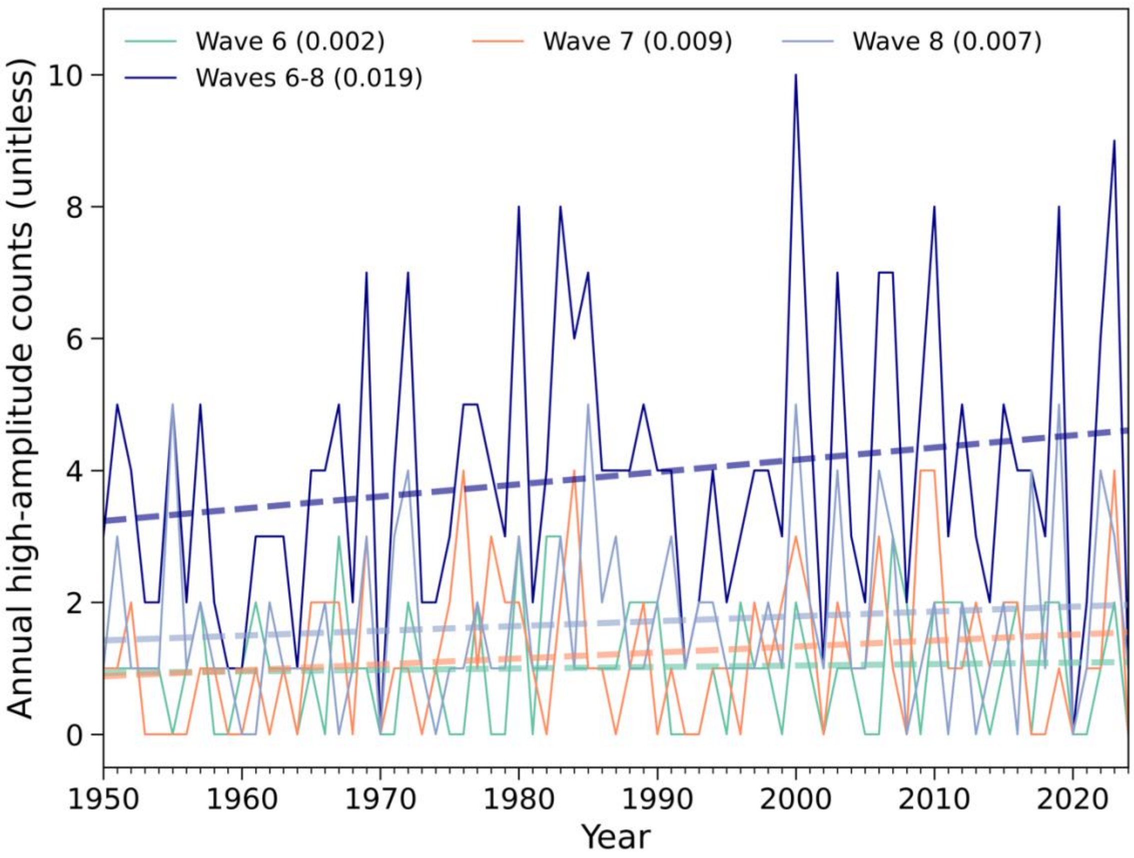

Fig. S2. Time series of high-amplitude event counts falling into zonal wavenumbers 6, 7, 8 individually and for the entire band 6–8 from 1950–2024. Linear trends for each time series are shown as thick dashed lines, with the corresponding slopes indicated in brackets within the legend. In no case are the trends statistically significant at the $p = 0.05$ level. A high-amplitude event is defined as a cluster of contiguous high-amplitude days (amplitude anomalies ≥ 1.5σ), and the annual counts represent the number of such events during each June-July-August (JJA) season.

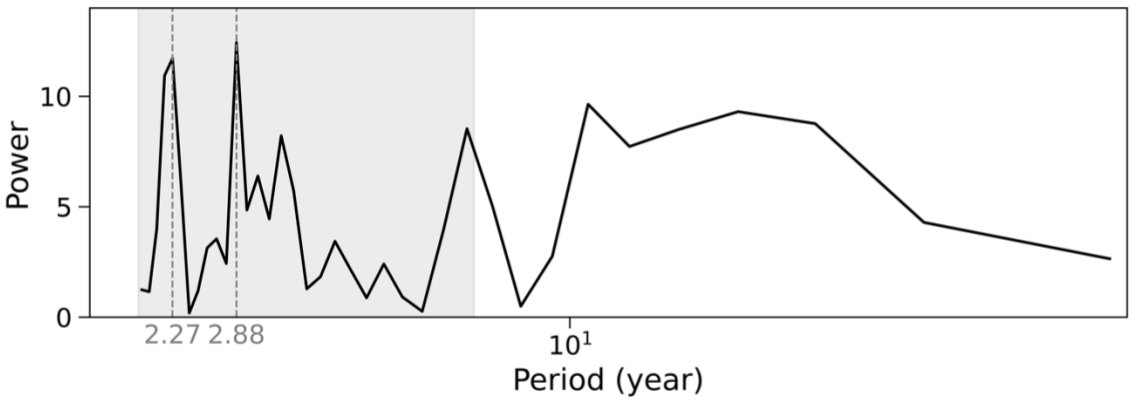

Fig. S3. Power spectral density analysis of the QRA time series (1950–2024). Prominent peaks in the power spectrum are marked by gray dashed lines, falling within the ENSO interannual band (2–7 years), highlighted by light gray shading.

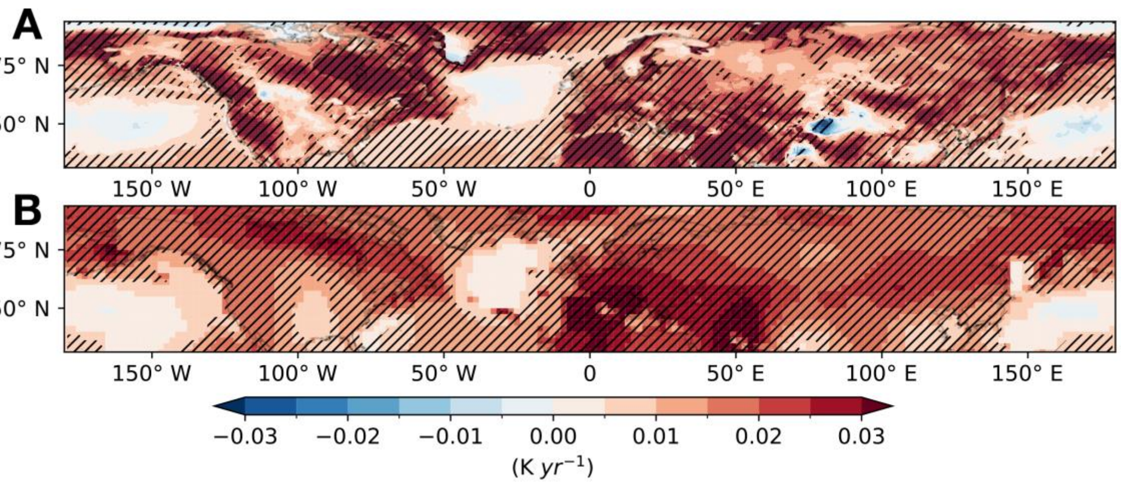

Fig. S4. Surface temperature anomalies trend over the overlapping period (1950–2016) for the reanalysis-derived QRA series and the QRA fingerprint series. (A) ERA5. (B) GISS. Hatching indicates that the trend is significant at the 95% level based on a two-sided Student *t*-test.

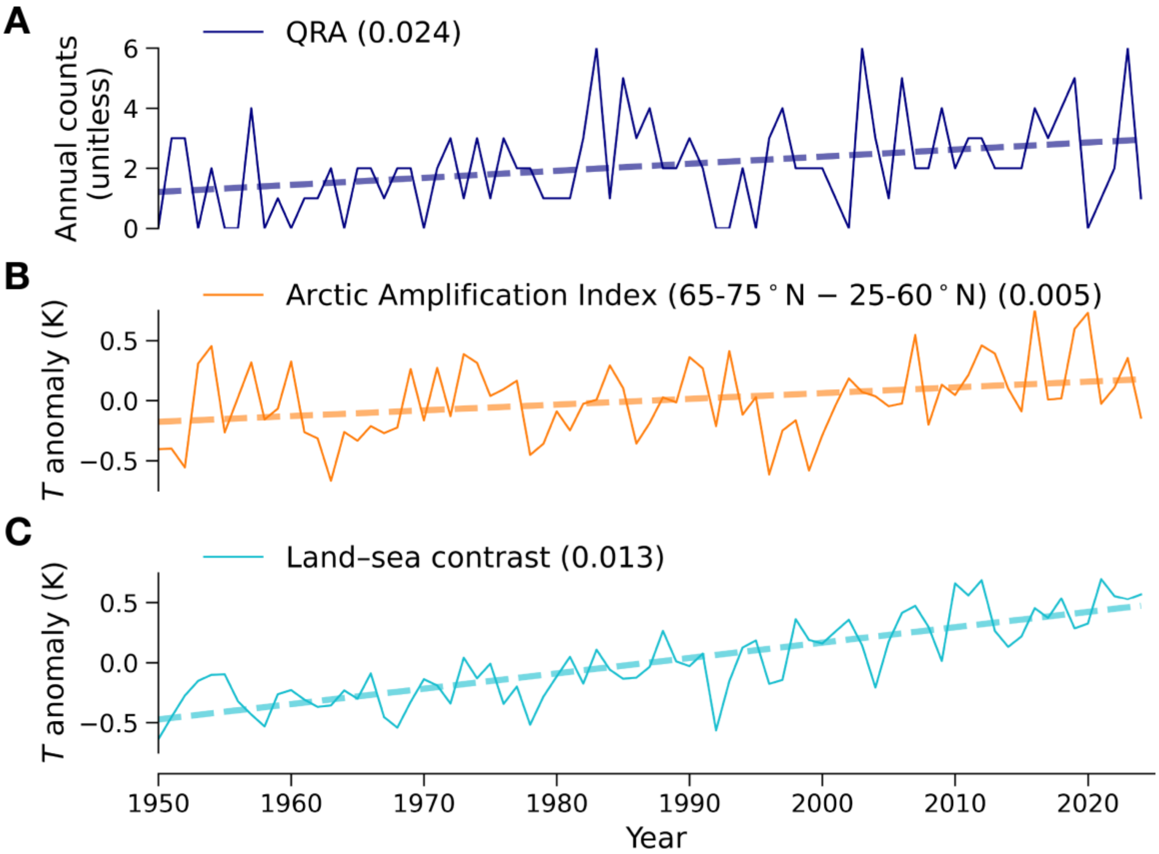

Fig. S5. Time series of boreal summer QRA event counts and associated boreal summer climate conditions over 1950–2024. (A) QRA event counts. (B) Arctic Amplification index, defined as the JJA zonal mean surface temperature difference between 65–75°N and 25–60°N. (C) Land–sea thermal contrast, defined as the difference between land and ocean temperature anomalies over the Northern Hemisphere, averaged for the boreal summer period. Linear trends for each series are shown as thick dashed lines, with the corresponding slopes indicated in brackets within the legend. All trends are statistically significant at the $p < 0.01$ level, as determined by both a two-tailed Student's t-test and a non-parametric Mann-Kendall test.

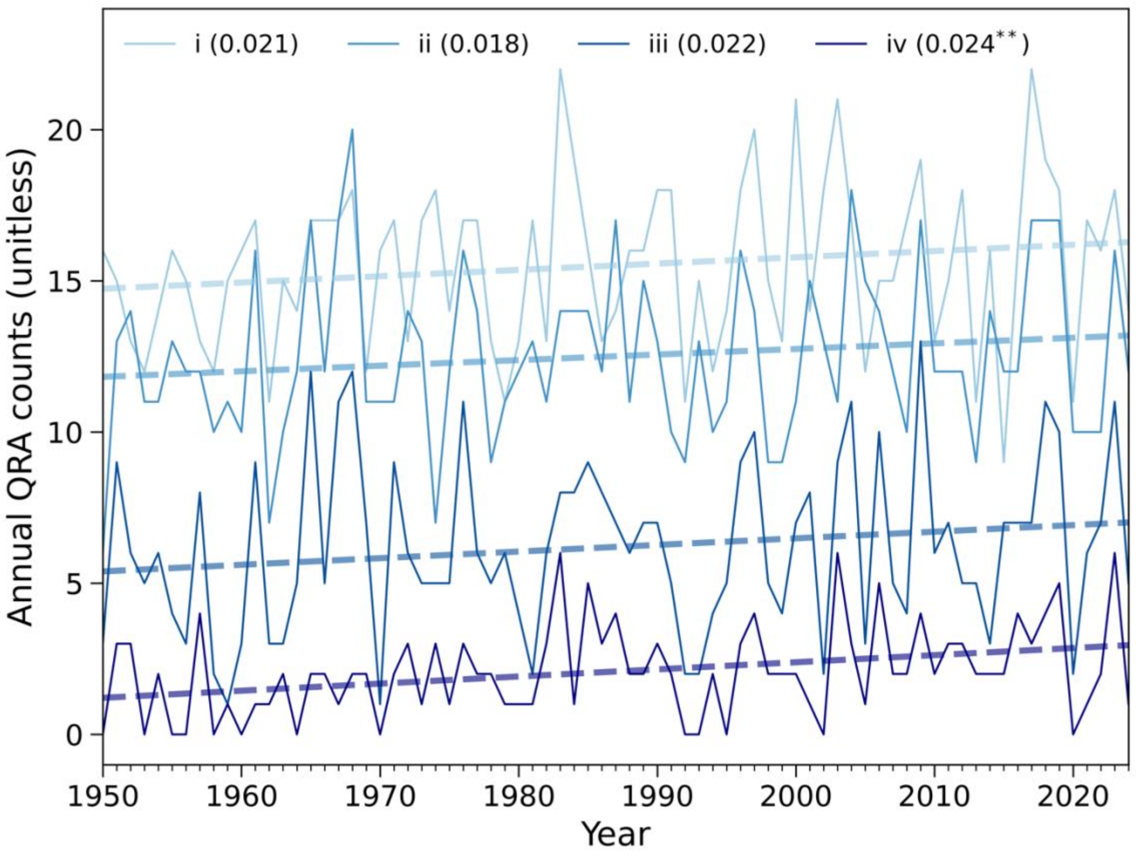

Fig. S6. Time series of boreal summer QRA event counts under varying detection conditions. Condition (i) requires the presence of a waveguide for a wave number $k \approx m$. Condition (ii) includes condition (i) plus a sufficiently strong effective forcing for wave number m. Condition (iii) includes conditions (i) and (ii) as well as the amplitude test that requires the observed wave amplitude falls within the range of $k = m \pm 0.2$. Condition (iv) builds upon conditions (i)–(iii) and additionally applies an amplitude filter, requiring amplitude to exceed 1.5 standard deviations above the 1950–2024 climatology. Condition (iv) serves as the primary criterion used throughout the main text, including the blue line shown in Fig. 1. Linear trends for each time series are shown as thick dashed lines, with the corresponding slopes indicated in brackets within the legend. A double asterisk (**) indicates significance at the 99% level.

Table S1. The years with a high frequency of QRA events and the occurrence of El Niño during the period 1950–2024. The former is defined as frequencies of no less than 3, with those exceeding 3 being boldfaced, while the latter is determined based on the Oceanic Niño Index (ONI) during the boreal winter, with extreme El Niño events (i.e., strong to very strong) boldfaced. The years where these events overlap are highlighted in italics.

Period	QRA	El Niño
1950s	*1951*, *1952*, ***1957***	*1951–52*, 1953–54, **_1957_–58**, 1958–59
1960s		1963–64, 1965–66, 1968–69, 1969–70
1970s	*1972*, 1974, *1976*	**_1972_–73**, *1976*–77, 1977–78, 1979–80
1980s	*1982*, ***1983***, **1985**, *1986*, ***1987***	***1982–83***, *1986-87*, *1987*–88
1990s	1990, 1996, ***1997***	**1991–92**, 1994–95, **_1997_–98**
2000s	***2003***, *2004*, ***2006***, ***2009***	2002–*03*, *2004*–05, *2006*–07, **_2009_–10**
2010s	2011, 2012, ***2016***, 2017, ***2018***, ***2019***	2014–15, **2015–_16_**, *2018–19*
2020s	***2023***	**_2023_–24**
